# Reduced glutathione and raffinose lengthens postharvest storage of cassava root tubers by improving antioxidant capacity and antibiosis

**DOI:** 10.1186/s12870-023-04466-7

**Published:** 2023-10-09

**Authors:** Haitian Fu, Ying Zhao, Jianqi Huang, Yanchun Luo, Zusheng Wei, Benchi Yu, Feng Wen

**Affiliations:** 1 Guangxi Subtropical Crops Research Institute, Nanning, 530001 P.R. China; 2 International Cooperation Base for Science and Technology of Cassava, Ministry of Science and Technology, Nanning, 530001 P.R. China

**Keywords:** Cassava, GR15231, Raffinose, Reduced glutathione, SC14, Storage tolerance

## Abstract

Cassava is an ideal food security crop in marginal and drought environment. However, the post-harvest storage of cassava is urgent problem to be resolved. In this study, the storage tolerant and non-tolerant cassava were screened by measuring the change of Peroxidase (POD), Superoxide dismutase (SOD), Catalase (CAT) and Malondialdehyde (MDA) in seven cultivars of cassava. Compared with other cultivars, the cultivar of SC14 showed the highest level of SOD, MDA and POD respectively at 0 day, 12 day and 9 day postharvest while exhibited lowest level of CAT at 0 day postharvest, indicating the strongest antioxidant capability and storage tolerance. In contrast, GR15231, termed as storage non-tolerance cultivars, showed lowest SOD and POD at 12 day and kept a relative high level of CAT at 12 day post-harvest. In addition, SC14 has higher level of starch and dry substance than GR15231. Mass spectrum was performed for SC14 and GR15231 to explore the key metabolites regulating the storage tolerance of cassava. The results showed that the expression of glutathione (reduced) and raffinose was significantly decreased at 12 day post-harvest both in tolerant SC14 and non-tolerant GR15231. Compared with GR15231, SC14 showed higher level of raffinose both at 0 and 12 day post-harvest, indicating that raffinose may be the potential metabolites protecting SC14 cultivar from deterioration post-harvest. Additionally, raffinose ratio of SC14a/SC14b was five times less than that of GR15231a/GR15231b, reflecting the slower degradation of raffinose in SC14 cultivar compared with GR15231 cultivar. In conclusion, the antioxidant microenvironment induced by reduced glutathione and higher level of raffinose in SC14 cultivar might be the promising metabolites to improve its antioxidant capacity and antibiosis and thus maintained the quality of Cassava root tubers.

## Introduction

Cassava is an important economic crop in tropical countries, such as Africa, Asia, Latin America, and the Caribbean [1]. Cassava is a plant that is enriched in carbohydrates and used as a bio-renewable resource and for many applications in industrial processes [2]. Because of its relative high productivity even under adverse climatic and nutrient-poor soil conditions in some tropical and subtropical regions, hundreds of million Africans depend on cassava as food, with more than 90% were produced in sub-Saharan Africain being used for fresh consumption and processed food [3, 4]. Besides, Cassava offers many different alternative applications as processed food, animal feed, starch, alcohol biofuel for vehicles etc. As countries develop, their demand for all these products increases dramatically [5, 6]. However, the storage tolerance of cassava post-harvest still is the urgent problem to be resolved to improve the high-efficient application of cassava.

Although cassava can tolerate droughts tress and has a high yield and starch content of root tuber, its root tuber has a very short shelf life and is very easy to rot after harvest compared with other root tuber crops. It usually begins to deteriorate 1 to 3 days after harvest [7, 8], which means that they have to be used immediately or processed into dry products [9]. The decay and deterioration will reduce the transparency of starch, affect the quality of starch, seriously affect the processing of starch and fuel ethanol, and cause huge economic losses to enterprises and farmers. Previous study showed that pruning the cassava stem, leaving about a 20 cm to 30 cm, three weeks before harvest could delay the onset of primary deterioration of the plant [10]. In recent years, efforts have been made to increase cassava production by improving agronomic practices and decreasing loss due to biotic and abiotic stress [11, 12]. However, rapid postharvest physiological deterioration of cassava storage roots reduces their market ability and limits the potential of this plant as a food and industrial crop. Therefore, the main goal of cassava breeding is to enhance storage tolerance and delay the decay and deterioration of cassava root tubers after harvest and improve their shelf-life.

In order to boost the storage tolerance of cassava, mass spectrum analysis of different cultivars of cassava were performed and the key regulators responding for the quick postharvest decay and deterioration were screened. In addition, the changes in antioxidant enzymes, starch and dry substance levels associated with changes in quality of cassava during postharvest storage were measured, and the differentiated substance and related metabolic pathways of the new cassava resistant to post harvest physiological deterioration were compared and analyzed. we comprehensively measured the level of antioxidant enzymes that regulating the deterioration in seven cultivars of cassava, and found that POD, SOD, CAT presented the highest level in SC14 cultivars and the lowest level in GR15231. In addition, SC14 and GR15231 cultivars respectively showed lowest and highest level of MDA compared with other six cultivars at 12 day post-harvest. SC14 showed higher level of starch and dry substance than GR15231. Then, we conducted UHPLC-Q Exactive HFX analysis for storage tolerant SC14 and non-tolerant GR15231cultivars and found that the antioxidant microenvironment induced by reduced glutathione and higher level of raffinose in SC14 cultivar might be the promising metabolites to improve its antioxidant capacity and antibiosis and thus maintained the quality of cassava root tubers.

## Materials and methods

### Plant materials

The materials used are from Nanning Cassava Germplasm Resources Nursery of Guangxi Subtropical Crops Research Institute. 3 varieties: SC14, NZ199, SC205; 4 lines: GR15231, 17–2, 17–15, W28. In February 2021, the spacing between plants and rows will be 0.8 m × 1.0 m, harvest it manually in December after 10 months, try to keep the cassava root tubers intact, and 50 kg cassava roots of each variety and line are placed on plastic greenhouse shelves. The samples were taken on the 0, 3, 6, 9 and 12 day after harvest. The head, middle, and tail parts of thre erandom cassava roots was collected. The collected cassava roots were chopped and mixed well. 200 g mixture was took every three days and stored at -80 ℃.

### Sample extraction

Following the slow thawing of the sample at 4℃, 100 mg of the sample was weighed. Subsequently, 100 ul of pre-cooled water was added to it. After vortexing for 60 s, 400 μL of a pre-cooled methanol–acetonitrile solution (1:1, v/v) was added, followed by another 60 s of vortexing. The sample was then subjected to low-temperature ultrasound for 30 min, repeated twice. It was then placed at -20℃ for 1 h to allow protein precipitation. Afterward, centrifugation was performed at 12000 rpm and 4℃ for 20 min to obtain the supernatant. The supernatant was subjected to vacuum drying and then re-dissolved in 200 μl of 30% acetonitrile (CAN). After vortexing and centrifuging at 14000 g and 4℃ for 15 min, the resulting supernatant was collected for testing.

### Instrument parameters

The data acquisition instrument system primarily comprises ultra-high performance liquid chromatography (UPLC, Vanquish, Thermo, USA) and high-resolution mass spectrometry (Q Executive HFX, Thermo, USA).

### Determination of cassava root starch content

On the 12 day, the content of starch in 5 kg SC14 and GR15231 was measured respectively, repeating 3 times. The harvested cassava tubers were weighed with the fresh cassava starch measuring instrument imported from Thailand, and the fresh cassava starch content was calculated according to the formula formulated by International Center for Tropical Agriculture. The formula: $\mathrm{P}= \left(210.8\times \frac{\mathrm{w}1}{\mathrm{w}1-\mathrm{w}2}-213.4\right)\times 100\mathrm{\%}$. (P = Cassava starch content; W1 = Weight of fresh cassava roots; W2 = Weight of fresh cassava roots in water).

### Determination of dry substance of cassava root

Three storage root samples were randomly selected from each variety (line). Each sample weighed 200 g fresh weight, cut into thin slices, and dried continuously to constant weight in a 60 °C oven. The dry matter content was expressed as the percentage of the dry weight of the storage root in the fresh weight.

### Liquid chromatographic parameters

Chromatographic column: Waters HSS T3 (100 * 2.1 mm, 1.8 μm); mobile phase: Phase A consists of a 0.1% formic acid water solution, while phase B comprises a 0.1% formic acid acetonitrile isopropanol mixture; flow rate: 0.3 mL/min; Column temperature: 40℃; Injection volume: 2 μL; elution gradient: 0.0–2.0 min (A/B, 90:10 V/V), 6.0–15.0 min (A/B, 40:60 V/V), 15.1–17.0 min (water/acetonitrile, 90:10 V/V). Throughout the analysis process, samples are stored in an automatic sampler at 4℃. To mitigate the impact of instrument detection signal fluctuations, a randomized sequence is employed for continuous sample analysis. Quality control (QC) samples are interspersed within the sample queue to monitor and assess the system's stability and the reliability of experimental data.

### UPLC Conditions

Sample extracts underwent analysis using a UPLC–Orbitrap-MS system (UPLC, Vanquish; MS, HFX). The analytical conditions were as follows: UPLC: column, Waters HSS T3 (50*2.1 mm, 1.8 μm); column temperature, 40℃; flow rate, 0.3 mL/min; injection volume, 2 μL; solvent system, water (0.1% acetic acid): acetonitrile (0.1% acetic acid); gradient program, 90:10 V/V at 0 min [13], transitioning to 90:10 V/V at 1.0 min, maintaining 90:10 V/V at 7.0 min, and concluding with 90:10 V/V at 9.0 min. The metabolic experiment using UPLC was conducted in four groups, with each group containing six replicates.

### LC–MS/MS analysis

HRMS data were acquired using a Q Exactive HFX Hybrid Quadrupole Orbitrap mass spectrometer equipped with a heated ESI source (Thermo Fisher Scientific) and employing the SIM MS acquisition methods. The ESI source parameters were configured as follows: spray voltage, -2.8 kV/3.0 kV; sheath gas pressure, 40 arb; auxiliary gas pressure, 10 arb; sweep gas pressure, 0 arb; capillary temperature, 320℃; and auxiliary gas heater temperature, 350℃ [14].

### Mass spectrum conditions

The primary and secondary spectra were collected using the Q Exactive HFX high-resolution mass spectrometry system from Thermo Company in the United States. The conditions of the electrospray ion source (ESI) were as follows: sheath gas at 40 psi, auxiliary air at 10 psi, ion spray voltage ranging from 2800 to 3000 V, a temperature of 350℃, and an ion transfer tube temperature of 320℃. The scanning mode included Full scan MS2 mode and positive/negative ion modes. The primary scanning range covered an m/z range of 70–1050 Da, while the secondary scanning range spanned 200–2000 Da. The primary resolution was set at 70,000, and the secondary resolution at 17,500.

### Data analysis process

Metabolomics data processing was performed using Progenesis QI software (Waters Corporation, Milford, USA). This involved baseline filtering, peak identification, integration, retention time correction, and peak alignment of the original data, resulting in a data matrix containing retention time, mass-to-charge ratio, and peak intensity information. Following data preprocessing, bioinformatics analysis was conducted, which included multidimensional statistical analysis performed using R software (PCA, PLS-DA, OPLS-DA) [15], differential metabolite screening (based on standard VIP > 1 and P < 0.05 criteria), correlation analysis of differential metabolites, KEGG pathway analysis [16–18], and other relevant analyses. Databases such as http://www.hmdb.ca/, https://metlin.scripps.edu/, as well as self-built databases, were utilized as primary data sources. Subsequently, data preprocessing steps were executed: 1) Retention of only non-zero variables that accounted for 80% of the total in any group of samples; 2) Total peak normalization and removal of variables with a relative standard deviation (RSD) of QC samples ≥ 30%; 3) Conversion of data into a log10 scale to create a data matrix for further analysis. Data analysis encompassed univariate statistical analysis (volcano plot), multidimensional statistical analysis, differential metabolite screening, correlation analysis of differential metabolites, KEGG pathway analysis, and more.

### Principal component analysis

PCA analysis was employed to identify prominent data elements and structures, reduce noise and redundancy, and decrease the dimensions of the original complex data. Additionally, PCA was used to identify and remove abnormal samples and evaluate the repeatability of quality control (QC) measures. Following dimension reduction analysis, samples were represented as relative coordinate points on the principal components PC1 and PC2. The distance between each coordinate point indicated the degree of similarity or dissimilarity between samples, with closer points denoting higher similarity. PCA analysis allowed for the observation of group separation trends, identification of potential outliers, and assessment of variability both between and within groups based on the original data. The primary parameter used to assess the quality of the PCA model was R2X. The PCA model parameters obtained through 7-fold cross-validation are presented in the table below. This value represents the extent to which the reduced-dimensional data interprets the original data. The closer R2X is to 1, the better the model. Generally, an R2X value greater than 0.5 indicates a good model.

### Multidimensional statistical analysis

The "data matrix" file was imported into R software for analysis. Initially, unsupervised principal component analysis (PCA) was employed to visualize the overall distribution of each sample and the degree of dispersion between groups. Subsequently, supervised (orthogonal) partial least squares discriminant analysis ((O)PLS-DA) was utilized to identify overall differences in metabolic profiles between groups and identify differential metabolites. In the OPLS-DA analysis, variables with variable importance in projection (VIP) greater than 1 were considered significant. To prevent overfitting, the model's fitting performance was evaluated through 200 permutation tests.

### OPLS-DA analysis

Orthogonal Partial Least Squares Discriminant Analysis (OPLS-DA) is a derivative algorithm of PLS-DA. This method utilizes partial least squares regression to establish a relationship model between metabolite expressions and sample categories, enabling the prediction of sample categories. OPLS-DA is an enhancement of Partial Least Squares Discriminant Analysis (PLS-DA) designed to filter out irrelevant noise variables that do not contribute to classification information, thereby improving the model's analytical capabilities and effectiveness. Unlike PLS, OPLS-DA has the ability to filter or disregard "noise" variables that are unrelated to the predicted variables. OPLS-DA segregates variables into two parts: the first part represents differences related to grouping, while the second part represents differences unrelated to grouping (orthogonal). OPLS-DA can effectively distinguish between these two parts, enhancing its ability to differentiate group differences and improving overall model effectiveness and analytical capabilities. Model evaluation parameters (R2Y, Q2) obtained through sevenfold cross-validation are presented in the table below. Typically, Q2 values exceeding 0.5 indicate a stable and reliable model, while Q2 values ranging from 0.3 to 0.5 indicate good model stability, and Q2 values below 0.3 suggest low model reliability.

### Differential metabolite screening

In PLS-DA analysis, the Variable Importance for the Projection (VIP) score is calculated to assess the influence of each metabolite's expression pattern on the classification and discrimination of sample groups, as well as its explanatory power. This aids in the identification of potential marker metabolites, typically with a VIP score greater than 1.0 serving as the screening criterion. Metabolites with VIP > 1 are typically considered as differential metabolites or potential markers [15]. A combination of the T-test and multivariate analysis using OPLS-DA was employed to identify differentially expressed metabolites between groups (while simultaneously considering VIP > 1 and a *p*-value < 0.05) and to conduct subsequent bioinformatics analysis.

### Volcanic map of differential metabolites

Univariate analysis is the simplest and most commonly employed method for analyzing experimental data. In the analysis of differential metabolites between the two groups of samples, two univariate analysis methods, Fold Change (FC) and T-test, were utilized and visualized using Volcano Plots. The screening criteria for identifying differential metabolites on the volcano plot were as follows: FC > 1.5 or FC < 1.5 with a *p*-value < 0.05.

### Differential metabolite clustering heatmap

To assess the validity of candidate metabolites and provide a more comprehensive and intuitive representation of the relationship between samples and the variations in metabolite expression patterns among different samples, the expression levels of qualitatively significant differential metabolites were utilized to perform hierarchical clustering within each sample group. This facilitated the precise identification of marker metabolites and the investigation of alterations in associated metabolic processes. Typically, when the selected candidate metabolites are appropriate and accurate, samples from the same group tend to cluster together during the clustering analysis. Metabolites clustered within the same group exhibit similar expression patterns, potentially indicating their involvement in closely related metabolic pathways.

### Enrichment analysis of differential metabolite KEGG pathway

The KEGG pathway database contains information related to metabolism, genetic information processing, environmental information processing, cell processes, biological systems, human diseases, and drug development. Our analysis was based on the KEGG database and associated website information, which can be found at http://www.genome.jp/kegg/pathway.html. The KEGG pathway enrichment analysis involved assessing the significance of metabolite enrichment for each pathway using Fisher's Exact Test. Pathways were analyzed individually, with the metabolic pathways relevant to the species or closely related species serving as the background. This analysis aimed to identify significantly impacted metabolic and signal transduction pathways. In general, a smaller *P*-value in the KEGG pathway enrichment results (*P* < 0.05) indicates a higher level of statistical significance in the enrichment. The number of differentially expressed metabolites within a KEGG pathway reflects, to some extent, the degree to which the biological treatment affects that pathway in the experimental design. Therefore, by considering these two factors, we selected metabolic or signal transduction pathways of interest and differentially expressed metabolites that significantly influence these pathways for subsequent biological experimental validation or mechanistic research.

### MetPA analysis of differential metabolites

KEGG pathway topology analysis primarily involves evaluating the relative importance of metabolites or biomolecules within a pathway using weighted scores based on the cyclic reaction structure and the relative positions of biomolecules. To facilitate comparisons between different pathways, the comprehensive score for each pathway was standardized to 1. The importance of each biomolecule was assessed by assigning it a weighted score based on its relative positional significance. The cumulative importance score for a given pathway was then calculated by aggregating the weighted scores of the matched metabolites. Higher scores indicate a greater influence on the pathway. In this analysis, the KEGG topology bubble diagram was employed to visualize the relative impact of differential metabolites on the pathway.

### Metabolite classification analysis

All metabolites identified in this study (including those identified in both positive and negative ion modes) were categorized and enumerated based on their chemical taxonomy attributes. The distribution of various metabolite categories is presented in the figure. Additionally, a categorized statistical chart of different metabolites within the comparison group is provided.

## Results

### The activity of antioxidant enzymes in different cultivar of cassava at different time-points after harvest

**Fig. 1:**
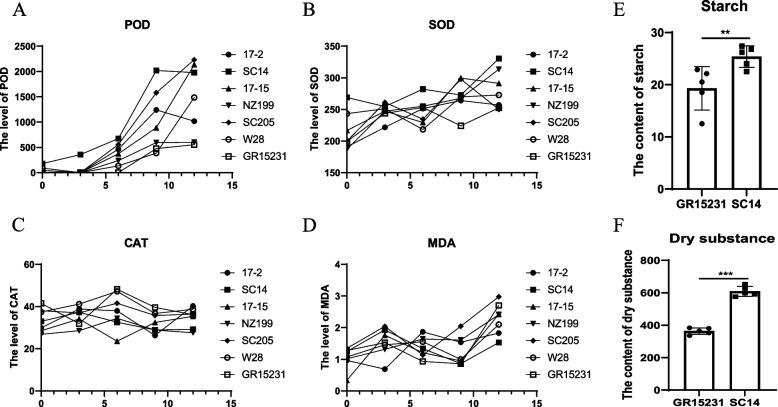
The activity of antioxidant enzymes in different cultivar of cassava at different time-points after harvest. **A**-**D** The level of POD (**A**), SOD (**B**), CAT (**C**) and MDA (**D**) in17-2, SC14, 17–15, NZ199, SC205, W28 and GR15231 cultivars at 0 day, 3 day, 6 day, 9 day and 12 day post-harvest. **E** The contents of starch in SC14 and GR15231 cultivars at 12 day post-harvest. **F** The measurement of dry substance in SC14 and GR15231 cultivars at 12 day post-harvest

To screen the cultivar of cassava with excellent storage tolerance, the enzymatic activity was analyzed among seven common cultivars, including 17–2, SC14, 17–15, NZ199, SC205, W28, GR15231. Previous studies showed that the level of antioxidant enzymes closely reflected the post-harvest storage tolerance of cassava. Therefore, we detected the level of four most important antioxidant enzymes for the roots storage, including Peroxidase (POD), Superoxide dismutase (SOD), Catalase (CAT) and Malondialdehyde (MDA). Compared with other cultivars, the cultivar of SC14 showed the highest level of SOD, MDA at 12 day and POD at 9 day postharvest while exhibited lowest level of CAT at 0 day postharvest (Fig. 1A, B, C). In contrast, the cultivar of GR15231 showed lowest SOD and POD at 12 day and kept a relative high level of CAT post-harvest (Fig. 1A, B, C, D). In addition, the contents of starch and dry substance in SC14 and GR15231 were detected, and the results showed that SC14 showed significantly higher level of starch and dry substance than GR15231, reflecting higher economic value of SC14 (Fig. 1E, F). Therefore, SC14 and GR15231 were assigned as the storage tolerant and non-tolerant Cassava cultivars respectively and were used subsequently for mass spectrum to identify the key regulators of storage capability.

### The grouping of tolerant and non-tolerant cassava cultivars and UHPLC-Q Exactive HFX analysis

To explore the key targets regulating the tolerance to storage, the cultivars of SC14 and GR15231 were chose and grouped them into tolerant and non-tolerant cassava respectively. To compared the dynamic change of potential targets, the tissues was collected at 0 day (SC14a and GR15231a) and 12 day (SC14b and GR15231b) post-harvest to UHPLC-Q Exactive HFX analysis, and six repeats were included in each group and time-points (Table 1). Totally, 587 different expressed metabolites (DEMs) were detected among the four groups, and 42, 33, 45 and 43 DEPs were identified for the comparation of SC14b_vs_SC14a, SC14a_vs_GR15231a, SC14b_vs_GR15231b and GR15231b_vs_GR15231a respectively (Table 2), indicating that the components of cassava would change significantly due to the difference of storage tolerance and storage time.

**Table 1:** The grouping of tolerant and non-tolerant cassava cultivars and UHPLC-Q Exactive HFX analysis

10-SC14	SC14a_1	0 day post harvest	10–15-23–1	GR15231a_1	0 day post harvest
10-SC14	SC14a-2		10–15-23–1	GR15231a_2	
10-SC14	SC14a_3		10–15-23–1	GR15231a_3	
10-SC14	SC14a_4		10–15-23–1	GR15231a_4	
10-SC14	SC14a_5		10–15-23–1	GR15231a_5	
10-SC14	SC14a_6		10–15-23–1	GR15232a_6	
22-SC14	SC14b_1	12 day post harvest	22–15-23–1	GR15231b_1	12 day post harvest
22-SC14	SC14b_2		22–15-23–1	GR15231b_2	
22-SC14	SC14b_3		22–15-23–1	GR15231b_3	
22-SC14	SC14b_4		22–15-23–1	GR15231b_4	
22-SC14	SC14b_5		22–15-23–1	GR15231b_5	
22-SC14	SC14b_6		22–15-23–1	GR15231b_6	

SC14 a: 0 day post-harvest; SC14 b: 12 day Post-harvest; GR15231a: 0 day post harvest; GR15231b: 12 day post-harvest. Six repeats were included in each group

**Table 2:** The differentially expressed metabolites in SC14 and GR15231 cassava at 0 and 12 day post-harvest

name	SC14b_vs_SC14a	SC14a_vs_GR15231a	SC14b_vs_GR15231b	GR15231b_vs_GR15231a
all_num	587	587	587	587
diff_num	42	33	45	43

Four comparation including:SC14b_vs_SC14a, SC14a_vs_GR15231a, SC14b_vs_GR15231b and GR15231b_vs_GR15231a were conducted

### The statistics of DEMs between tolerant and non-tolerant cassava by chemical taxonomy

**Fig. 2:**
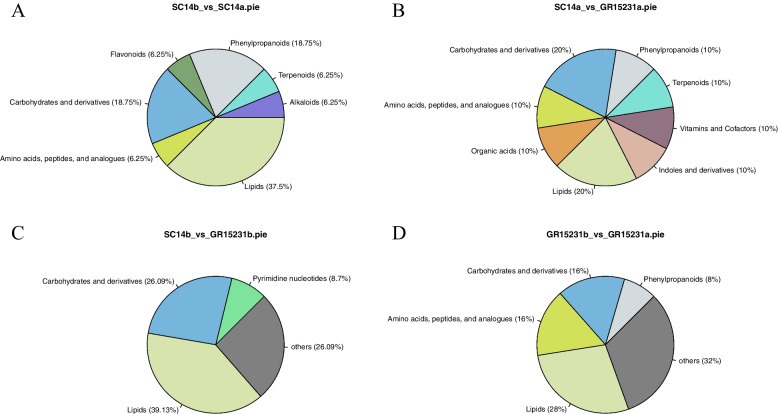
The statistics of DEMs between tolerant and non-tolerant cassava by Chemical Taxonomy. **A**-**D** The distribution of metabolites in SC14 and GR15231 cultivar at 0 and 12 day post-harvest. Four comparation, including SC14b_vs_SC14a (**A**), SC14a_vs_GR15231a (**B**), SC14b_vs_GR15231b (**C**), and GR15231b_vs_GR15231a (**D**), were conducted

To overall delineate the effects of different cassava varieties and different storage time on the change of components contents, such as carbohydrates and derivatives, amino acids, peptides and analogues, Lipids and phenylpropanoids, chemical taxonomy analysis for all groups were performed according to the classification. In the storage tolerant cultivar of SC14a, the main DEMs were enriched in the taxonomy of lipids (37.5%), carbohydrates and derivatives (18.75%) and phenylpropanoids (18.75%) between the roots of 0 day and 12 day post-harvest (Fig. 2A). Whereas, we not only observed the consistent enrichment of lipids (20%), carbohydrates and derivatives (20%) and phenylpropanoids (10%) but also the newly enriched organic acids (10%), vitamins and cofactors (10%), and indoles and derivatives (10%) in SC14a vs GR15231a (Fig. 2B). In contrast, the enrichment of lipids (28%), carbohydrates and derivatives (16%) and amino acids, peptides and analogues (16%) and phenylpropanoids (18.75%) were observed between the roots of 0 day and 12 day post-harvest both in the comparation of GR15231b vs GR15231a and SC14b vs GR15231b. However, no microelement, such as organic acids, vitamins and cofactors and indoles and derivatives were obviously enriched in storage non-tolerant GR15231a and GR15231b groups compared with tolerant SC14a and SC14b respectively (Fig. 2C, D). Taken together, we proposed that the enriched organic acids, vitamins and cofactors and indoles might be responsible for the storage tolerance of SC14 cultivars.

### KEGG analysis of enriched signaling pathways in tolerant and non-tolerant cassava post-harvest

**Fig. 3:**
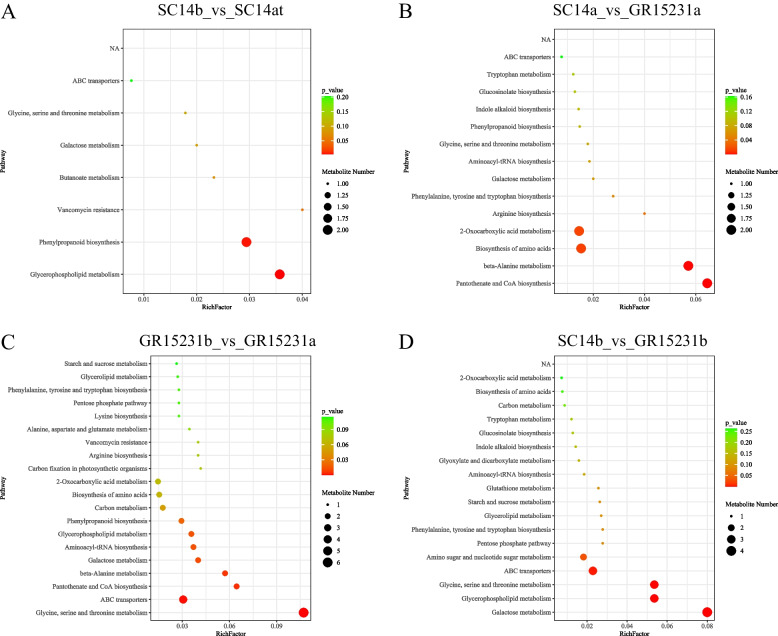
KEGG analysis of enriched signaling pathways in SC14 and GR15231 cassava post-harvest. **A**-**D** The enrichment of signaling pathways in SC14 and GR15231 cultivar at 0 and 12 day post-harvest. Four comparation, including SC14b_vs_SC14a (**A**), SC14a_vs_GR15231a (**B**), GR15231b_vs_GR15231a (**C**), and SC14b_vs_GR15231b (**D**), were conducted

To investigate the key metabolism pathways mastering the storage tolerance in SC14 cassava, we performed KEGG analysis for DEMs in different groups. The pathway of phenylpropanoid biosynthesis and glycerophospholipid metabolism were significantly enriched in SC14a vs SC14b and GR15231a vs GR15231b, indicating the time-dependent decrease of these two pathways activity in both SC14 and GR15231 cultivars (Fig. 3A, C). In addition, these two pathways were the top 2 enriched in SC14a vs SC14b while it only showed slight enrichment in SC14a vs GR15231a (Fig. 3A, B). In contrast, compared with SC14a, GR15231a showed lower activity of phenylpropanoid biosynthesis and glycerophospholipid metabolism but higher level of Beta-Alanine metabolism, pantothenate and CoA biosynthesis at 0 day post-harvest, which reflecting the fast decomposition of carbohydrates and lipids in GR15231 (Fig. 3C). Consistently, the pathways that responsible for Cassava deterioration, including amino sugar and nucleotide sugar metabolism, glycerophospholipid metabolism and galactose metabolism, were even higher enrichment at 12 day in SC14b vs GR15231b (Fig. 3D). Therefore, we inferred that the metabolites participating in phenylpropanoid biosynthesis and glycerophospholipid metabolism could be promising targets to protect the storage tolerance of SC14 cultivar.

### Sample correlation analysis for different cassava cultivars

**Fig. 4:**
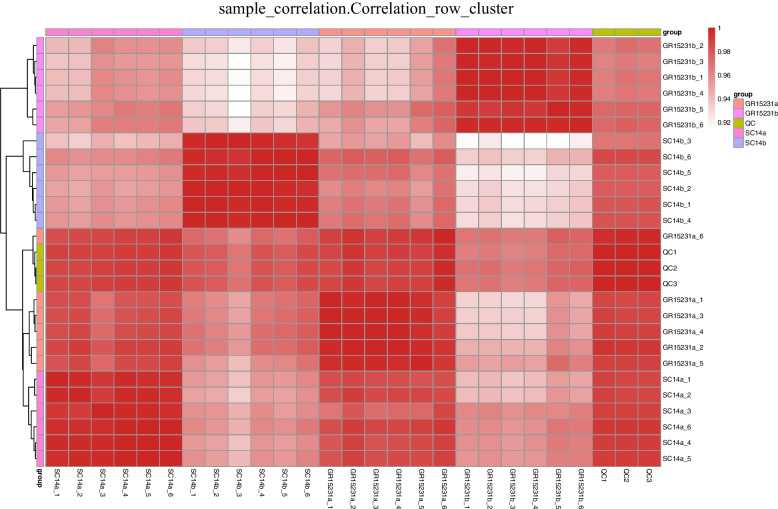
Correlation analysis based on DEMs in SC14 and GR15231 cassava post-harvest. The samples correlation inter-group and inner-groups were clustered and presented by heatmap for SC14 and GR15231 cultivar at 0 and 12 day post-harvest. Four groups were included, including SC14a, SC14b, GR15231a and GR15231b were conducted

In order to clearly analyze the metabolic differences of different varieties of cassava at different storage time points, the sample correlation analysis according to the DEMs in SC14 and GR15231 cassava were performed. Firstly, we observed that the intra-group differences of samples in all groups and QC groups were very small and showed good repeatability, indicating the reliability of our data. Importantly, SC14a and GR15231a are clustered together, while SC14b and GR15231b are clustered together, but only see relatively weak correlation clustering between SC14a and SC14b was observed. In conclusion, the metabolic profiles of SC14 and GR15231 were affected by cassava cultivars and storage time, and the storage time exert stronger influence on metabolic spectrum (Fig. 4).

### The PCA analysis of samples in tolerant and non-tolerant cassava

**Fig. 5:**
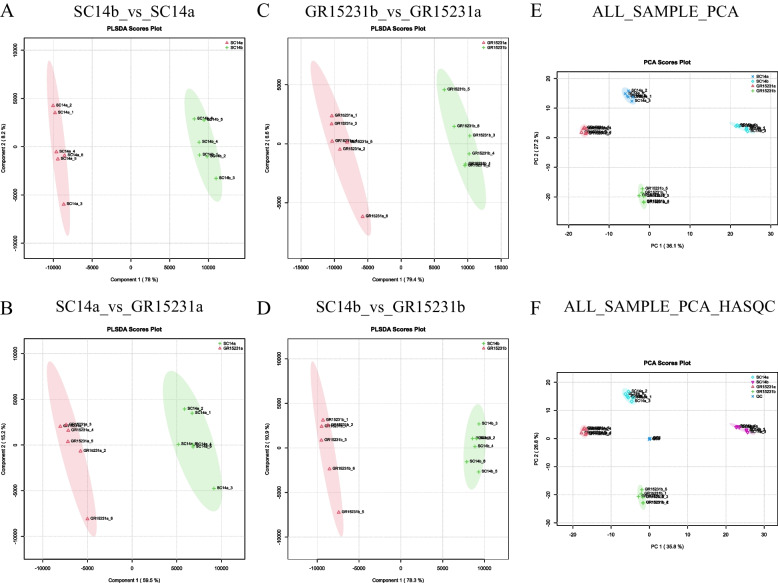
The PCA analysis for samples in SC14 and GR15231 cassava with different timepoint post-harvest. **A**-**D** The distribution of samples in SC14 and GR15231 cultivar at 0 and 12 day post-harvest were clustered. Four comparation, including SC14b_vs_SC14a (**A**), SC14a_vs_GR15231a (**B**), (**C**), and GR15231b_vs_GR15231a (**C**), SC14b_vs_GR15231b, were conducted. **E**–**F** The PCA (**E**) and HASQC (**F**) PCA analysis for all groups of SC14 and GR15231cultivars. SC14a, 0 day post-harvest; SC14b, 12 day post-harvest; GR15231a, 0 day post-harvest; GR15231b, 12 day post-harvest

To testify variability the inter-group difference and difference within groups, the principal component analysis (PCA) of metabolites was conducted. The results showed that the six repeat in both SC14a and SC14b were efficiently clustered together while there was a significant variability between samples in SC14a and SC14b, indicating the obvious effect of storage time on the properties of SC14 cultivar (Fig. 5A-D). Consistently, the obvious cluster of inter-group samples and apparent segregation within groups of GR15231 cultivar were observed (Fig. 5E-F). Taken together, we concluded that the inter-group difference is qualified for both SC14a and SC14b cultivar and the metabolites spectrum were obvious affected by the length of storage post-harvest.

### The heatmap analysis and Volcano Plot of DEPs in tolerant and non-tolerant cassava

**Fig. 6:**
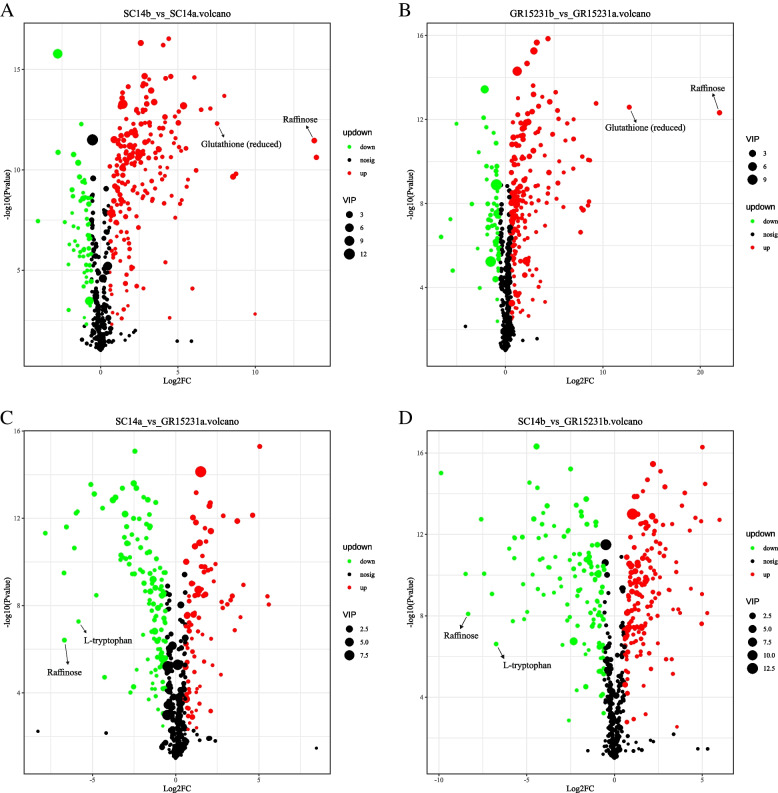
The Volcano plot of DEMs in SC14 and GR15231 cassava post-harvest. **A**-**D** The samples correlation inter-group and inner-groups were clustered and presented by heatmap for SC14 and GR15231 cultivar at 0 and 12 day post-harvest. Four groups were included, including SC14a, SC14b, GR15231a and GR15231b were conducted. The change of glutathione (reduced), L-tryptophan and raffinose were notified in figures

**Fig. 7:**
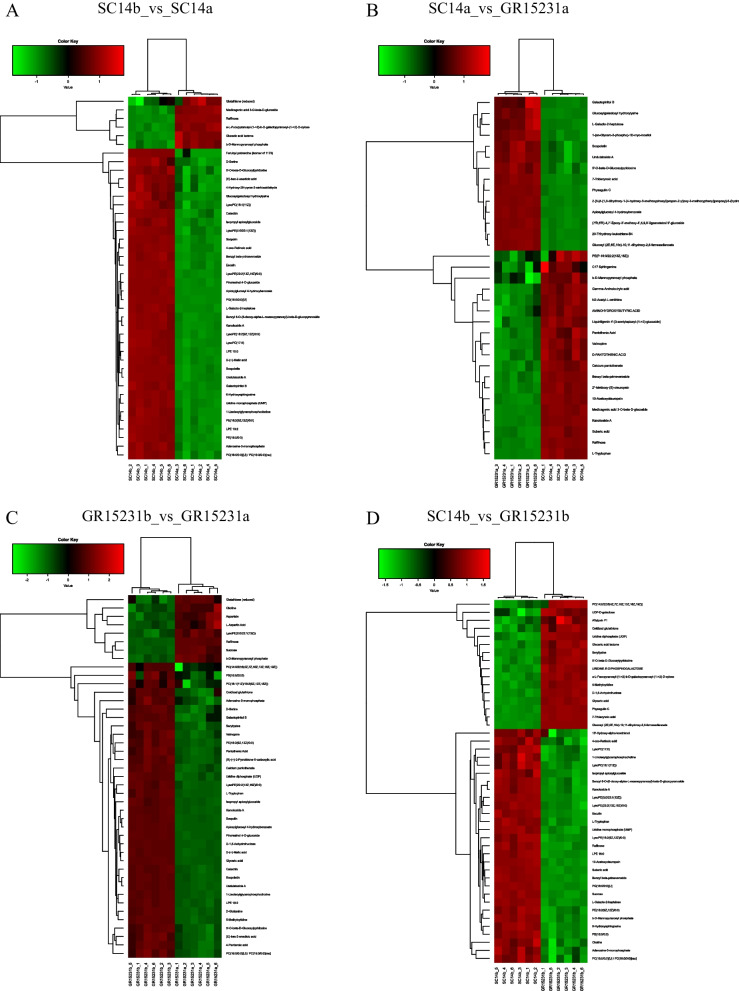
Heatmaps of DEMs in SC14 and GR15231 cassava for different times post-harvest. **A**-**D** The enrichment of signaling pathways in SC14 and GR15231 cultivar at 0 and 12 day post-harvest. Four comparation, including SC14b_vs_SC14a (**A**), SC14a_vs_GR15231a (**B**), GR15231b_vs_GR15231a (**C**), and SC14b_vs_GR15231b (**D**), were conducted

**Fig. 8:**
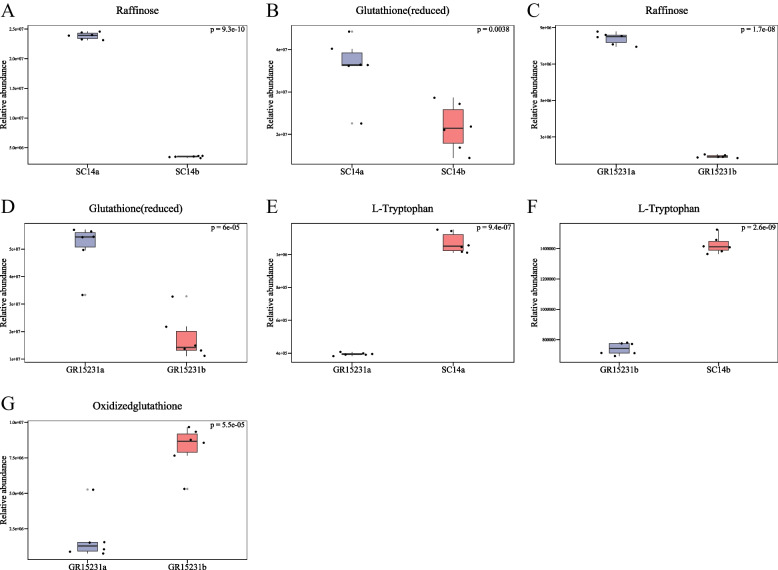
The level of glutathione and raffinose in SC14 and GR15231 cassava with different timepoint post-harvest. **A**-**B** The level of raffinose (**A**), glutathione (**B**) of samples in SC14a and SC14b. **C**-**D** The level of raffinose (**C**), glutathione (**D**) in GR15231a and GR15231b cultivars. **E**–**F** The level of L-tryptophan in GR15231a vs SC14a and GR15231b vs SC14b. **G** The level of oxidized glutathione in GR15231a and GR15231b

**Fig. 9:**
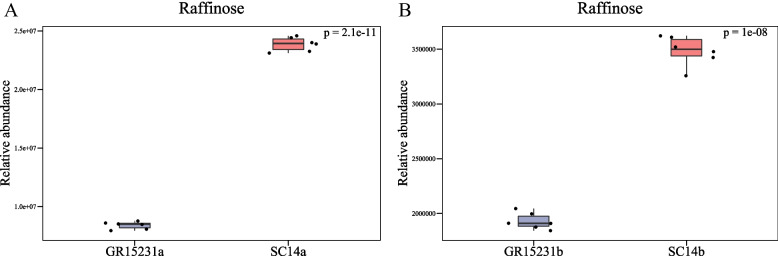
The level of raffinose and L-Tryptophan in SC14 and GR15231 cassava with different timepoint post-harvest. **A** The level of raffinose in SC14a and GR1531a cultivars. **B** The level of raffinose in SC14b and GR1531b cultivars

In order to analyze the differential metabolite between the two groups of samples, the Fold Change and T-test were comprehensively analyzed to draw the Volcano Plot and heatmaps of the differential metabolite. The results showed that the expression of glutathione (reduced) and raffinose in SC14a and GR15231a was significantly higher than SC14b and GR15231b, indicating its critical role for the storage tolerance in both tolerant SC14a and non-tolerant GR15231 cultivars (Fig. 6A-D, Fig. 7A-D, 8A-D). Additionally, we found the significant higher level of L-tryptophan in SC14a and SC14b respectively compared with GR15231a and GR15231b and the significant increase of oxidized glutathione in GR15231b compared with GR15231a, indicating the lower oxidative stress in SC14 cultivar and the time-dependent increase of oxidative stress in GR15231 (Fig. 8E-G). Importantly, a significant decreasing expression of raffinose were observed in the comparation both in SC14a vs GR15231a and SC14b and GR15231b (Fig. 9A, B), indicating that raffinose may be the potential metabolites protecting SC14 cultivar from deterioration post-harvest. Finally, heatmap analysis for DEMs of all groups were conducted, and consistently found that the raffinose ratio of SC14a/SC14b was five times less than that of GR15231a/GR15231b, reflecting the slower degradation of raffinose in SC14 cultivar compared with GR15231. Taken together, the antioxidant microenvironment induced by reduced glutathione and higher level of raffinose might be the promising metabolites to promote the storage tolerance of cassava.

## Discussion

The preservation of cassava post-harvest has always been a difficult problem to solve [11]. Post-harvest physiological deterioration of cassava occurs when blue or brown spots appeared, and microbial invasion, such as aflatoxin and penicillium, and oxidative stress induced by metabolites would cause decay and deterioration of cassava after 5 to 7 days post-harvest, which caused great loss of product and seriously restricted the annual supply of cassava and its comprehensive utilization after production [19–21]. Therefore, the screening and breeding of cassava with great storage tolerant cassava could be a promising strategy to solve this dilemma. In this work, we comprehensively measured the level of antioxidant enzymes that regulating the deterioration in seven cultivars of Cassava, and we found that POD, SOD, CAT presented the highest level in SC14 cultivars and the lowest level in GR15231. In addition, SC14 and GR15231 cultivars respectively showed lowest and highest level of MDA compared with other six cultivars at 12 day post-harvest. SC14 showed higher level of starch and dry substance than GR15231. Then, we performed UHPLC-Q Exactive HFX analysis for storage tolerant SC14 and non-tolerant GR15231 and found that the antioxidant microenvironment induced by reduced glutathione and higher level of raffinose in SC14 cultivar might be the promising metabolites to promote its storage tolerance.

The high level of antioxidant mediator in SC14 cultivar contributed to the storage tolerance. Previous evidence indicated that ROS-scavenging enzymes were activated as responses to the physiological stress and played an important role in delaying the physiological deterioration process in various plants [7, 22, 23]. High contents of hydrogen peroxide and guaiacol peroxidase activity in cassava roots can be used as potential biomarkers of post-harvest deterioration [24]. Enhanced reactive oxygen species scavenging by overproduction of superoxide dismutase and catalase delays postharvest physiological deterioration of cassava storage roots [25]. In this study, the expression of glutathione (reduced) in SC14a and GR15231a was significantly higher than SC14b and GR15231b, indicating its critical role for the storage tolerance in both tolerant SC14a and non-tolerant GR15231 cultivars. In addition, SC14a cultivar had higher level of reduced glutathione than GR15231a. Therefore, the antioxidant microenvironment by glutathione may be related to the high storage tolerance of SC14 compared with GR15231 cultivar.

Raffinose might be a novel metabolism to depress cassava post-harvest deterioration. Invasion of microorganisms, such as moulds and aflatoxins and penicillium seriously affects the storage tolerance of cassava post-harvest [21, 26]. Previous evidence showed that raffinose could efficiently resist fungi and bacteria infection. Ham et al. showed that raffinose inhibits streptococcus mutans biofilm formation by targeting Glucosyltransferase [27]. In addition, the raffinose family oligosaccharides were capable to act as antioxidants [28]. Kim et al. showed that raffinose could inhibit Pseudomonas aeruginosa biofilm formation via binding to LecA and decreasing cellular cyclic diguanylate levels, colony morphology, matrix formation, and swarming motility, and reduced the concentration of the second message and cyclic diguanylate by increased activity of cyclic diguanylate specific phosphodiesterase [29]. In our study, the expression of raffinose in SC14a and GR15231a was significantly higher than SC14b and GR15231b, indicating its critical role for the storage tolerance in both tolerant SC14a and non-tolerant GR15231 cultivars. Importantly, both SC14a and SC14b showed higher expression of raffinose than GR15231a and GR15231b respectively, reflecting raffinose may be the potential metabolites protecting SC14 cultivar from deterioration post-harvest. In addition, consistently found that the raffinose expression ratio of SC14a/SC14b was five times less than that of GR15231/GR15231b, implying the slower degradation of raffinose in SC14 cultivar compared with GR15231.

In conclusion, the antioxidant microenvironment induced by reduced glutathione and the manipulation of raffinose might be the promising metabolites to promote the storage tolerance of cassava.

## Data Availability

The datasets used and/or analysed during the current study available from the corresponding author on reasonable request.

## References

[1] Okogbenin E, Setter TL, Ferguson M, Mutegi R, Ceballos H, Olasanmi B, Fregene M (2013). Phenotypic approaches to drought in cassava: review. Eur J Front Physiol.

[2] Onabolu AO, Oluwole OS, Bokanga M, Rosling H (2001). Ecological variation of intake of cassava food and dietary cyanide load in Nigerian communities. Eur J Public Health Nutr.

[3] Sayre R, Beeching JR, Cahoon EB, Egesi C, Fauquet C, Fellman J, Fregene M, Gruissem W, Mallowa S, Manary M, Maziya-Dixon B, Mbanaso A, Schachtman DP, Siritunga D, Taylor N, Vanderschuren H, Zhang P (2011). The BioCassava plus program: biofortification of cassava for sub-Saharan Africa. Eur J Annu Rev Plant Biol.

[4] Lobell DB, Gourdji SM (2012). The influence of climate change on global crop productivity. Eur J Plant Physiol.

[5] Martinez-Burgos WJ, Sydney EB, Medeiros ABP, Magalhaes AI, De Carvalho JC, Karp SG, Vandenberghe LPDS, Letti LAJ, Soccol VT, Pereira GVDM (2021). Agro-industrial wastewater in a circular economy: Characteristics, impacts and applications for bioenergy and biochemicals. Eur J Bioresour Technol.

[6] Rueangsan K, Kraisoda P, Heman A, Tasarod H, Wangkulangkool M, Trisupakitti S, Morris J (2021). Bio-oil and char obtained from cassava rhizomes with soil conditioners by fast pyrolysis. Eur J Heliyon.

[7] Reilly K, Bernal D, Cortes DF, Gómez-Vásquez R, Tohme J, Beeching JR (2007). Towards identifying the full set of genes expressed during cassava post-harvest physiological deterioration. Eur J Plant Mol Biol.

[8] Bayoumi SAL, Rowan MG, Blagbrough IS, Beeching JR (2008). Biosynthesis of scopoletin and scopolin in cassava roots during post-harvest physiological deterioration: the E-Z-isomerisation stage. Eur J Phytochemistry.

[9] Zidenga T, Leyva-Guerrero E, Moon H, Siritunga D, Sayre R (2012). Extending cassava root shelf life via reduction of reactive oxygen species production. Eur J Plant Physiol.

[10] Ravi V, Aked J (1996). Review on tropical root and tuber crops. II. Physiological disorders in freshly stored roots and tubers. Eur J Crit Rev Food Sci Nutr.

[11] Sonnewald U, Fernie AR, Gruissem W, Schläpfer P, Anjanappa RB, Chang SH, Ludewig F, Rascher U, Muller O, Doorn AMV, Rabbi IY, Zierer W (2020). The Cassava Source-Sink project: opportunities and challenges for crop improvement by metabolic engineering. Eur J Plant J.

[12] Ceballos H, Hershey C, Iglesias C, Zhang X (2021). Fifty years of a public cassava breeding program: evolution of breeding objectives, methods, and decision-making processes. Eur J Theor Appl Genet.

[13] Wang Z, Wang L, Huang H, Li Q, Wang X, Sun Q, Li N (2022). In vitro antioxidant analysis of flavonoids extracted from Artemisia argyi stem and their anti-inflammatory activity in lipopolysaccharide-stimulated RAW 264.7 macrophages. Food Chem.

[14] Xie M, Luo Y, Gao T, Li R (2023). Investigation on the lubrication component and mechanism for a biolubricant isolated from the agro-waste resource of *Codonopsis pilosula*. Sci Total Environ.

[15] Yang Q, Ding J, Feng X, Zhong X, Lan J, Tang H, Harwood W, Li Z, Guzmán C, Xu Q, Zhang Y, Jiang Y, Qi P, Deng M, Ma J, Wang J, Chen G, Lan X, Wei Y, Zheng Y, Jiang Q (2022). Editing of the starch synthase IIa gene led to transcriptomic and metabolomic changes and high amylose starch in barley. Carbohydr Polym.

[16] Kanehisa M, Goto S (2000). KEGG: kyoto encyclopedia of genes and genomes. Nucleic Acids Res.

[17] Kanehisa M (2019). Toward understanding the origin and evolution of cellular organisms. Protein Sci.

[18] Kanehisa M, Furumichi M, Sato Y, Kawashima M, Ishiguro-Watanabe M (2023). KEGG for taxonomy-based analysis of pathways and genomes. Nucleic Acids Res.

[19] Reilly K, Gomez-Vasquez R, Buschmann H, Tohme J, Beeching JR (2004). Oxidative stress responses during cassava post-harvest physiological deterioration. Eur J Plant Mol Biol.

[20] Ono LT, Silva JJ, Dona S, Martins LM, Iamanaka BT, Fungaro MHP, Pitt JI, Taniwaki MH (2021). Aspergillus section Flavi and aflatoxins in Brazilian cassava (*Manihot esculenta* Crantz) and products. Eur J Mycotoxin Res.

[21] Ekpakpale DO, Kraak B, Meijer M, Ayeni KI, Houbraken J, Ezekiel CN (2021). Fungal Diversity and Aflatoxins in Maize and Rice Grains and Cassava-Based Flour (Pupuru) from Ondo State. Nigeria. Eur J Fungi (Basel).

[22] Apel K, Hirt H (2004). Reactive oxygen species: metabolism, oxidative stress, and signal transduction. Eur J Annu Rev Plant Biol.

[23] Uarrota VG, Moresco R, Carlos SE, Laurita BZ, Eduardo dCN, Enilto dON, Martins PLA, Miguel R, Marcelo M. The role of ascorbate peroxidase, guaiacol peroxidase, and polysaccharides in cassava (*Manihot esculenta* Crantz) roots under postharvest physiological deterioration. Eur J Food Chem. 2016;197(Pt A): 737-746. 10.1016/j.foodchem.2015.11.025 26617011

[24] Jebara S, Jebara M, Limam F, Aouani ME (2005). Changes in ascorbate peroxidase, catalase, guaiacol peroxidase and superoxide dismutase activities in common bean (Phaseolus vulgaris) nodules under salt stress. Eur J Plant Physiol.

[25] Xu J, Duan X, Yang J, Beeching JR, Zhang P (2013). Enhanced reactive oxygen species scavenging by overproduction of superoxide dismutase and catalase delays postharvest physiological deterioration of cassava storage roots. Eur J Plant Physiol.

[26] Ono LT, Silva JJ, Soto TS, Doná S, Iamanaka BT, Fungaro MHP, Taniwaki MH (2022). Fungal communities in Brazilian cassava tubers and food products. Eur J Int J Food Microbiol.

[27] Ham SY, Kim HS, Cha E, Lim T, ByunY PHD (2022). Raffinose inhibits streptococcus mutans biofilm formation by targeting glucosyltransferase. Eur J Microbiol Spectr.

[28] ElSayed AI, Rafudeen MS, Golldack D (2014). Physiological aspects of raffinose family oligosaccharides in plants: protection against abiotic stress. Eur J Plant Biol (Stuttg).

[29] Kim HS, Cha E, Kim Y, Jeon YH, Olson BH, Byun Y, Park HD (2016). Raffinose, a plant galactoside, inhibits Pseudomonas aeruginosa biofilm formation via binding to LecA and decreasing cellular cyclic diguanylate levels. Eur J Sci Rep.

